# Individual and environmental variables related to outdoor walking among older adults: Verifying a model to guide the design of interventions targeting outdoor walking

**DOI:** 10.1371/journal.pone.0296216

**Published:** 2024-01-10

**Authors:** Yixiu Liu, Nancy M. Salbach, Sandra C. Webber, Ruth Barclay

**Affiliations:** 1 Department of Community Health Sciences, University of Manitoba, Winnipeg, Manitoba, Canada; 2 Department of Physical Therapy, University of Toronto, Toronto, Ontario, Canada; 3 KITE Research Institute, Toronto Rehabilitation Institute—University Health Network, Toronto, Ontario, Canada; 4 Department of Physical Therapy, University of Manitoba, Winnipeg, Manitoba, Canada; South China Normal University, CHINA

## Abstract

**Objective:**

To estimate the relationships between individual and environmental variables and outdoor walking (OW) in older adults with OW limitations through verifying a conceptual model.

**Methods:**

Baseline data from 205 older adults participating in a randomized trial of a park-based OW program were analyzed using structural equation modeling. We evaluated a three latent factor model: OW (accelerometry and self-report); individual factors (balance; leg strength; walking self-confidence, speed and endurance; mental health; education; income; car access); and environmental factors (neighbourhood walkability components).

**Results:**

Mean age was 75 years; 73% were women. Individual factors was significantly associated with OW (β = 0.39, *p* < .01). Environmental factors was not directly associated with OW but was indirectly linked to OW through its significant covariance with the individual factors (β = 0.22, *p* < .01). The standardized factor loadings from the individual factors on walking self-confidence and walking capacity measures exceeded 0.65.

**Conclusions:**

Better walking capacity and more confidence in the ability to walk outdoors are associated with higher OW in older adults. Better neighbourhood walkability is indirectly associated with more OW. The conceptual model demonstrates an individual and environment association; if the capacity of the individual is increased (potentially through walking interventions), they may be able to better navigate environmental challenges.

## Background

Walking outdoors can lead to substantial benefits in older adults, such as better physical and mental health, improved health-related quality of life, lower risk of chronic health conditions, and lower risk of mortality [1–5]. However, individual characteristics such as reduced balance and leg strength [6], insufficient walking self-efficacy, high fear for personal safety [7], and inadequate knowledge regarding using walking aids [8] can limit outdoor walking (OW). Additionally, environmental characteristics, including poor social support, street-related hazards (e.g., traffic, curbs, walking and/or cycling lanes), access to services (e.g., benches, public bathrooms), and neighbourhood safety, also contribute to reductions in OW [9–12]. Therefore, it is important to consider both individual and environmental characteristics and their association with OW to guide the development of interventions to increase OW in older adults.

Some previous studies focused on the effect of either individual or environmental variables on OW. The researchers in those studies assumed the variables were independent and applied traditional statistical methods, such as multivariate linear regression [11], logistic regression [6, 10, 12] and Cox proportional hazard model [13]. Other researchers developed multilevel models to account for the dependence of environmental variables but ignored the dependence of individual variables [9, 14, 15]. However, multiple individual variables such as components of walking capacity (i.e., comfortable and fast gait speed, walking endurance, leg strength, balance capacity, and self-reported walking self-efficacy) are correlated [16–18], as are different environmental variables such as land use diversity and residential density [19, 20]. Although the correlation among individual/environmental variables may not be high enough to cause collinearity problems when using aforementioned methods, it is important to take the correlation into consideration when building a model.

Structural equation modeling (SEM), a powerful approach to analyze complex relationships among a number of variables [21], can be used to examine how individual and environmental variables interact, and their influence on OW. Using SEM, the correlation among observed variables can be accounted for by assuming a latent construct to be an underlying factor [21]. One study in this area applied an SEM approach and found that leisure time physical activities were significantly associated with individual level variables (i.e., perceived exercise benefits and barriers, and exercise self-efficacy) while not significantly associated with environmental level variables [22]. Another study applied SEM and found that individual factors (i.e., self-efficacy, perceived pros and cons of physical activity, and social support) had direct influences on physical activity, while the environmental factors affected physical activity indirectly through individual and social factors [23]. However, these two studies emphasized psychological characteristics from an individual perspective but did not consider measured physical abilities. Moreover, the two studies focused on populations (i.e., Chinese and Japanese adults, respectively), which have significantly different cultural values and attitudes towards physical activity and have different physical activity levels compared to Canadians [24, 25]. For example, a study conducted in the US in 2005 found that whites, Hispanics and Koreans were more likely to participate in physical exercises such as walking, jogging, running, and bicycling etc. when visiting a park compared to African Americans, Chinese, and Japanese [26]. A study conducted in Scotland found that black and other non-white minority adults were less likely than white adults to report participating in outdoor recreation every week [27].

**Fig 1:**
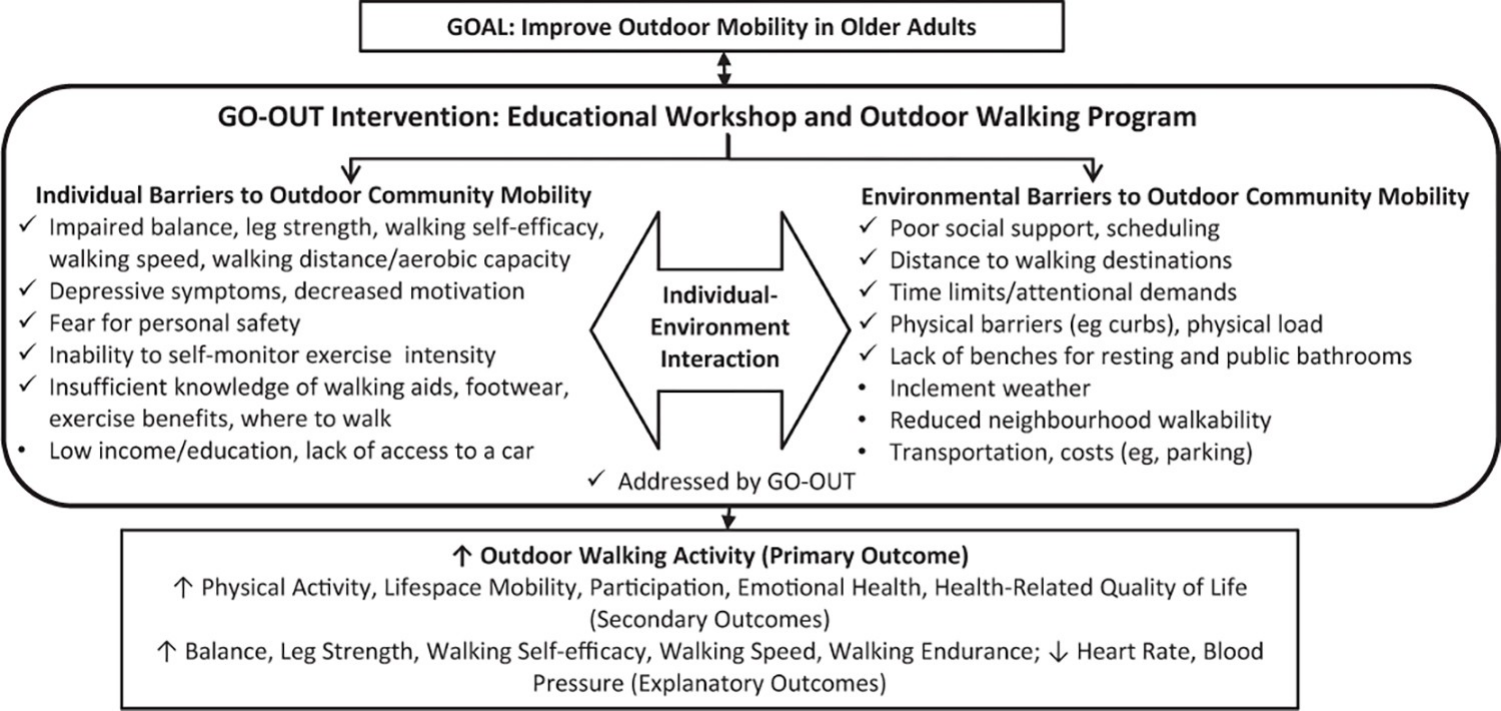
Conceptual framework for the GO-OUT intervention. Note: This figure is reprinted from the Fig 1 by Salbach NM, Barclay R, Webber SC, et al. A theory-based, task-oriented, outdoor walking programme for older adults with difficulty walking outdoors: protocol for the Getting Older Adults Outdoors (GO-OUT) randomised controlled trial. BMJ Open 2019;9:e029393. doi: 10.1136/bmjopen-2019-029393.

The Getting Older Adults Outdoors (GO-OUT) study focused on the evaluation of the effect of a theory-based, task-oriented, park-based walking program targeted at increasing OW among older adults with self-reported difficulties in walking outdoors [28]. The conceptual framework (Fig 1) for the GO-OUT study [28] described the relationships between individual and environmental characteristics, and their association with OW; it was used to help design the study intervention to improve OW and potentially further improve health outcomes [28]. This conceptual framework, however, has not been tested.

Many physiotherapists work with older adults who live in the community, and support older adults in living active, healthy lives. In physiotherapy, theory can be used to influence and describe clinical practice and alternately, clinical practice experiences may be used to adapt theories [29]. A theoretical conceptual framework of OW for older adults could be used by physiotherapists in determining important areas of assessment and intervention for older adults wishing to improve OW in the community. Additionally, there is not a clear understanding of how to design interventions or community programs to increase OW [30], therefore, conceptual models can help us with intervention design in both research and community programs.

Physiotherapists and physical activity trainers may work with features of the outdoor physical environment, such as curbs or hills, and incorporate practice of these terrain aspects into an outdoor walking intervention [31–34]. They may also incorporate aspects of the social environment, such as social support, by providing group-based interventions [32–34]. The overall purpose of this study was to evaluate the variables related to OW as depicted in the GO-OUT conceptual framework among older adults with OW limitations. The objectives were: 1) to estimate the association between individual and environmental variables and OW in older adults, 2) to verify a conceptual model of OW, which was developed to guide interventions targeting OW among older adults [28].

## Methods

### Design and study sample

A secondary analysis of the baseline data from the GO-OUT randomized controlled trial (RCT) study was performed. The trial was registered on ClinicalTrials.gov, number NCT03292510. The GO-OUT RCT aimed to evaluate the effect of a theory-based, task-oriented, park-based walking program on promoting OW among older adults with self-reported difficulties walking outdoors, such as difficulties in walking over uneven ground, speeding up to cross the street, walking briskly for 30 minutes without a rest, or lacking confidence or motivation to walk outdoors for exercise. The protocol [28] and pilot study for the GO-OUT RCT [34], as well as multiple sub-studies [35–38] have been published. Detailed information about eligibility criteria, recruitment, outcomes, interventions, and data collection for the GO-OUT RCT were described in the protocol paper [28]. In summary, participants were community-dwelling older adults aged 65 years or older with self-reported difficulty in OW, recruited using community-based strategies, from Edmonton, Winnipeg, Toronto and Montreal, Canada. Participants attended an eight-station active walking workshop and then were randomized to the 10-week task-oriented outdoor walk group program or a 10-week telephone weekly reminders program [28]. Evaluations were completed at baseline, with follow-ups at 3 months, 5.5 months and 12 months. The data collected at the baseline evaluation prior to randomization were used in this study. In total, 205 people participated in the baseline evaluation. The baseline data were collected from May to July of 2018 and from April to June of 2019. Ethics Approval (HS24988 (H2021:226)) was received from the Health Research Ethics Board (HREB) of the University of Manitoba for this secondary analysis.

### Measures

#### Outdoor walking variables

The primary outcome was OW measured using device-based and self-reported approaches. The device-based measure was the number of minutes per week spent in OW calculated based on data captured by an accelerometer (ActiGraph GT3X+ activity monitor [ActiGraph, Pensacola, Florida, USA]) and a global positioning system (GPS) device (Qstarz BT-Q1000XTA-GPS Travel Recorder). ActiGraph monitors have demonstrated good reliability (intraclass-correlation coefficients (ICC) > 0.8), high inter-instrument reliability (ICC > 0.95), and good validity [39–42]. The location accuracy of Qstarz GPS has been shown to be 3 meters [1]. Participants wore the GT3X+ and QstarzBT devices on a belt around the waist during waking hours for eight days. A valid wear day was defined as a minimum wear time of 10 hours and we required at least four valid days of combined accelerometer and GPS data for individuals’ data to be included in analysis [43]. ActiLife6 software (version 6.13.3; ActiGraph LLC) was used to initialize the GT3X+ monitors to collect data at 100Hz. The GPS devices were set to collect data every 10s (Qstarz QTravel software, Qstarz International Co., Ltd.). Accelerometer data were downloaded using the low frequency extension filter in ActiLife6 which is recommended for people with slower walking speeds to increase sensitivity to lower intensity movements [44, 45]. A walking bout was defined as a period of activity ≥ 5 min in duration with cadence ≥ 40 steps/min, allowing a maximum of 1 min below threshold [46]. A custom R program (https://www.r-project.org) was used to detect start and end times of walking bouts in 60s epoch accelerometer files. Location of activity during the identified walking bouts was then manually checked in the GPS data by importing the corresponding latitude and longitude data points time-synchronized with the walking bouts into Google Earth Pro version 7.3.2 (https://www.google.com/earth/versions/#earth-pro). If the participant’s location during the walking bout time was in a park setting (e.g., outdoor path, golf course), a neighborhood setting (residential area) or on a sidewalk of a city street (non-residential area), then the walking was deemed to have occurred outdoors, and the duration of the walking bout was included in the OW minutes per week.

OW was also measured by the total score of five items adapted from the self-reported Community Health Activities Model Program for Seniors (CHAMPS) questionnaire to measure outdoor walking (CHAMPS-OUTDOORS) in older adults. The unit was hours spent in OW in a week. The validity of scores using CHAMPS-OUTDOORS to measure OW in older adults was examined using data from the GO-OUT study [35]. The reliability of the original CHAMPS is fair (ICC ranges from 0.79 to 0.85) to measure physical activity among older adults [47].

#### Individual variables

We included ten variables/items to measure the individual (latent) factors according to the GO-OUT conceptual framework [28].

***Balance capacity*** was assessed using the 14-item Mini Balance Evaluation Systems test (mini-BESTest) [48]. The total score can range from 0 to 28 with each item scored from 0 to 2 [48]. Higher scores indicate higher balance. Excellent reliability (ICC for inter-rater reliability ≥ 0.91) and good validity have been shown [49, 50].

***Leg strength*** was measured using the 30-second sit-to-stand test [51]. For this test, the number of sit-to-stands completed in 30 seconds is documented. High reliability (ICC ≥ 0.84) and excellent validity have been reported [52, 53].

***Walking self-efficacy*** was evaluated using the 22-item ambulatory self-confidence questionnaire (ASCQ) [54]. Each item is scored from 0 to 10 [54]. The mean of item-level scores is used to determine the total score. Higher scores indicate higher self-confidence. This questionnaire has been shown to be reliable (ICC = 0.92) and has acceptable validity [54].

***Walking speed*** was assessed using the 10-meter walk test [55] performed at a ***comfortable*** pace and ***fast*** pace. Walking speed in m/sec at a comfortable pace and fast pace were derived using the time in seconds to walk the middle 10 meters of a 14-meter walkway. Excellent reliability (ICC > 0.9) and construct validity have been reported [56–58].

***Walking endurance*** was measured using the 6-minute walk test (6MWT) [59] with a 30-meter straight walkway. Distance in meters walked in 6 minutes was documented. Studies have shown excellent test-retest reliability (ICC = 0.95) and validity of this test [60, 61].

***Mental health*** was evaluated using the emotional well-being scale in the Research ANd Development-36 (RAND-36) questionnaire [62]. The score was the average of the five items which was calculated according to the scoring instructions [63], ranging from 0 to 100. The construction of the survey and its psychometric properties have been studied [62, 64].

***Education***, ***access to a car***, and ***income*** were also included as individual variables.

### Environmental variables

***The neighbourhood environment walkability scale (NEWS)*** questionnaire is the most common questionnaire used to measure the perceived environment for physical activity research in older adults [65, 66]. The subscales from NEWS were assumed as variables that contributed to the environmental (latent) factor [19]. The NEWS questionnaire consists of 13 subscales including eight multi-item variables (i.e., A: residential density, B: land-use mix-diversity, C: land-use access, D: street connectivity, E: infrastructure and safety for walking, F: aesthetics, G: traffic hazards, and H: crime) and five single-item variables (i.e., I: lack of parking, J: lack of cul-de-sacs, K: hilliness, L: physical barriers, and N: social interaction while walking). Generally, higher scores of subscales B, C, D, E, F, I, J and N indicate more walkability of the environment, higher scores of subscales G, H, K and L indicate less walkability, and higher scores of subscale A indicate higher density. The scoring has been validated using confirmatory factor analysis [19, 20]. Fear related to personal safety was proposed as one variable under the individual characteristic component of the conceptual framework. This variable was, in fact, measured by the crime subscale from the NEWS questionnaire. Therefore, it was treated as one of the variables to measure the environmental factors in this study. A sensitivity analysis was conducted to compare the goodness-of-fit of the measurement model for the individual factors with and without fear related to personal safety as one observed variable.

### Statistical analysis

Data were analyzed using R version 1.2.5033 for Windows. We adopted the *lavaan* package in R for SEM modeling although other popular and powerful software such as Mplus is available. We chose R not only because R is freely available, but, more importantly, because *lavaan* also supports the WLSMV approach, which is recommended when having categorical indicators, and uses the same parameter estimation procedures compared to Mplus [67, 68]. Summary statistics, including mean, standard deviation (SD), median (25th and 75th percentiles) and range for continuous variables and frequencies and percentages for categorical variables, were used to describe the distribution of all variables. Variances of continuous variables were used to check the ill-scaled covariance matrix problem which happens when the variance of one variable is more than 100 times the variance of another variable [69]. The variables were rescaled through multiplying or dividing by a constant to make the variances more similar if the ill-scaling problem existed. Correlation-coefficient matrices for all variables were calculated to check correlations among observed variables and to identify collinearity. Polychoric, polyserial, and Pearson approaches were used to calculate the correlation coefficients for pairs of ordinal variables, pairs of ordinal and continuous variables and pairs of continuous variables, respectively.

**Fig 2:**
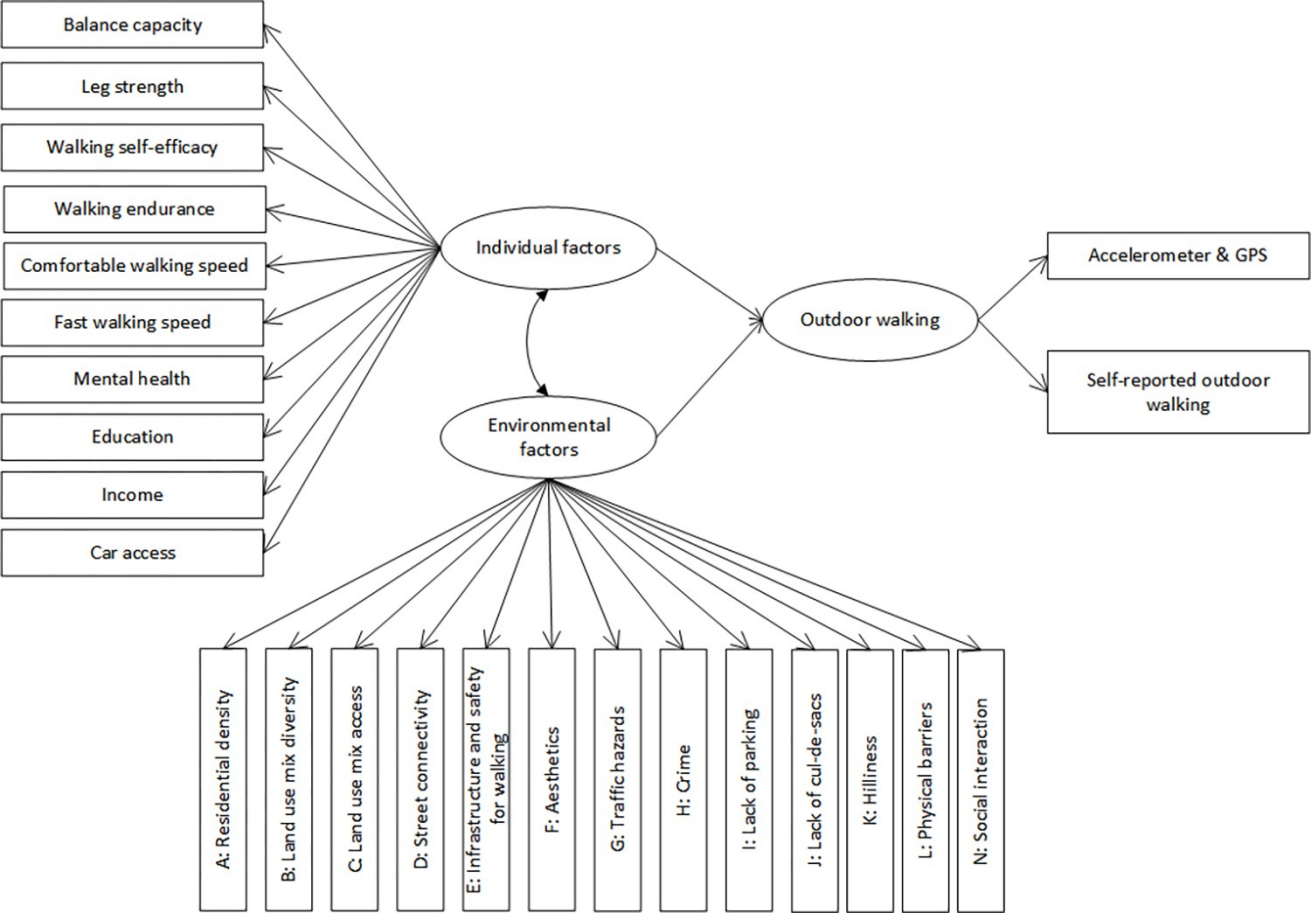
Initial structural equation model proposed based on the conceptual framework.

The SEM approach is useful for examining complex relationships among latent factors and observed variables [21]. Latent factors are constructs that cannot be measured directly but can be represented by a variety of observed variables which reflect the construct. In this study, the initially proposed SEM based on the conceptual framework is shown in Fig 2. The two measurement models in the initially proposed SEM were fit to determine whether the two latent factors–individual factors and environmental factors–in the SEM were properly constructed. The coefficients from latent factors to observed variables are called factor loadings. Standardized factor loadings estimate the correlation between the latent factor and observed variables ranging from -1 to 1. Values that are closer to 1 or -1 indicate stronger association. Then, two modified measurement models were developed according to the results of the initial measurement models. Finally, an SEM based on the two modified measurement models was built to estimate the path coefficients of the individual factors and the environmental factors to OW. The correlation of the two OW measurements used in the GO-OUT study, CHAMPS-OUTDOORS and accelerometry-GPS, is moderate (r = 0.33) [35, 70]. It is possible that the two measurements reflect different aspects of OW. Therefore, the OW in the SEM was treated as a latent factor with two observed variables–device-based measurement by accelerometry-GPS and self-reported measurement by the CHAMPS-OUTDOORS questionnaire. To further justify, one model that used device-based measurement for OW was fit and compared to the model that assumed an underlying latent factor for the two measurements for OW.

During data collection, a 10-meter walkway (instead of a 30-meter walkway) was used to administer the 6MWT with 11 participants. Therefore, a sensitivity analysis was conducted, wherein the analysis was performed with and without data from these 11 participants, to examine the effect of this protocol deviation on the results.

The weighted least square mean and variance (WLSMV) estimator was applied for parameter estimation. WLSMV is a robust approach for SEM with categorical variables or nonnormal variables [71, 72]. Delta parameterization was used to make the model identified through constraining the variances of latent factors to one to make the model identified. Missing data were addressed by pairwise deletion [68]. The full information maximum likelihood method was not used in this study because this method was not supported when using WLSMV approach. The model goodness-of-fit was assessed by relative indices including robust chi-square test and comparative fit index (CFI) and absolute index–root mean square error of approximation (RMSEA) and its 90% confidence interval (CI) [69]. A significant result (i.e., p value < 0.05) of the *χ*^2^ test indicates that the data do not support the hypothesized model. However, more weight was given to other goodness-of-fit indices because the *χ*^2^ test is very sensitive to sample size [69]. Larger values of CFI indicate better fit. The rule of thumb is that a CFI larger than 0.9 indicates a reasonable fit [69]. RMSEA measures the difference between conceptual and data-implied models; a value less than 0.05 indicates a good model fit while less than 0.08 indicates a reasonable fit [69]. To compare nested models when one model is a submodel of the other, chi-square difference test was used; a significant result supports the model with smaller chi-square test statistics [69].

## Results

Table 1 presents the descriptive statistics for participant characteristics and observed variables. Our sample consisted of 205 participants with baseline data collected prior to randomization. The mean age was 75 ± 7 years (range: 60 to 94 years) and 150 (73%) were females. The majority of participants were *able to have some money left over at the end of the month* (159 (78%)), followed by *just enough to make ends meet* (31 (15%)), while only three people had *not enough to make ends meet* (2%). Therefore, the two categories–*just enough to make ends meet* and *not enough to make ends meet* were merged in the data analysis.

**Table 1:** Participant characteristics (n = 205).

Characteristics	Categories, units, or score range	n (%)	Mean ± SD	Median (25, 75 percentile)	(Min, Max)
**Age**	Years	204	75 ± 7	73 (69, 79)	(60, 94)
**Sex**	Male	55 (27)			
** **	Female	150 (73)			
**Marital status**	Divorced/separated	31 (15)			
	Married	99 (48)			
	Single	26 (13)			
** **	Widowed	48 (23)			
**Education**	Less than secondary	15 (7)			
	Some post secondary	37 (18)			
	Completed secondary (grade 9–12)	26 (13)			
	Completed college (in Quebec—CEGEP)	35 (17)			
	Completed university (bachelor degree)	64 (31)			
** **	Graduate program (e.g., MSc, PhD)	28 (14)			
**Income**	Not enough to make ends meet	3 (2)			
	Just enough to make ends meet	31 (15)			
** **	Some money left over	159 (78)			
**Outdoor walking—CHAMPS**	hours per week	202	2.8 ± 3.6	1.8 (0.5, 3.5)	(0, 20.3)
**Outdoor walking—accelerometry+GPS**	hours per week	176	0.69 ± 0.95	0.36 (0.00, 0.89)	(0.00, 4.41)
**Balance capacity**	0–28 points	204	20 ± 5	22 (17, 24)	(4, 28)
**Leg strength**	# sit to stands	204	8 ± 4	9 (6, 10)	(0, 20)
**Walking self-efficacy**	0–10 points	203	7.9 ± 1.7	8.3 (7.0, 9.2)	(1.5, 10.0)
**Walking speed—comfortable pace**	m/s	204	1.06 ± 0.23	1.06 (0.91, 1.21)	(0.41, 1.67)
**Walking speed—fast pace**	m/s	204	1.40 ± 0.32	1.41 (1.18, 1.63)	(0.63, 2.18)
**Walking endurance**	meters walked in 6 minutes	202	353.6 ± 98.5	360.0 (286.2, 417.6)	(0.0, 583.0)
**Mental health**	0–100 points	202	76.1 ± 15.4	76.0 (68.0, 88.0)	(20.0, 100.0)
**Car access**	No	26 (13)			
** **	Yes	179 (87)			
**Variables from NEWS**				
**A: Residential density**	173–865 points	202	286.0 ± 131.4	221 (177.0, 373.8)	(102.0, 654.0)
**B: Land-use mix-diversity**	1–5 points	201	2.3 ± 0.8	2.3 (1.7, 3.0)	(0.9, 4.4)
**C: Land-use access**	1–4 points	200	2.8 ± 0.9	2.8 (2.3, 3.5)	(1.0, 4.0)
**D: Street connectivity**	1–4 points	200	2.9 ± 0.7	3.0 (2.3, 3.7)	(1.0, 4.0)
**E: Infrastructure and safety for walking**	1–4 points	200	2.9 ± 0.6	3.0 (2.6, 3.4)	(1.0, 4.0)
**F: Aesthetics**	1–4 points	199	3.1 ± 0.6	3.2 (2.8, 3.6)	(1.7, 4.0)
**G: Traffic hazards**	1–4 points	205	2.1 ± 0.6	2.2 (1.7, 2.5)	(1.0, 3.8)
**H: Crime**	1–4 points	205	1.7 ± 0.6	1.5 (1.3, 2.0)	(1.0, 4.0)
**I: Lack of parking**	Strongly disagree	82 (40)			
	Somewhat disagree	51 (25)			
	Somewhat agree	51 (25)			
	Strongly agree	15 (7)			
**J: Lack of cul-de-sacs**	Strongly disagree	35 (17)			
	Somewhat disagree	36 (18)			
	Somewhat agree	51 (25)			
	Strongly agree	77 (38)			
**K: Hilliness**	Strongly disagree	127 (62)			
	Somewhat disagree	46 (22)			
	Somewhat agree	19 (9)			
	Strongly agree	9 (4)			
**L: Physical barriers**	Strongly disagree	138 (67)			
	Somewhat disagree	38 (19)			
	Somewhat agree	17 (8)			
	Strongly agree	8 (4)			
**N: Social interaction while walking**	Strongly disagree	20 (10)			
	Somewhat disagree	31 (15)			
	Somewhat agree	91 (44)			
	Strongly agree	59 (29)			

Regarding the two observed variables for OW, the mean number of hours participants spent walking outdoors per week measured using the CHAMPS-OUTDOORS was 2.8 (SD = 3.6), ranging from 0 to 20.3 while the mean number of hours participants spent walking outdoors per week measured by accelerometry + GPS was 0.69 (SD = 0.95), ranging from 0 to 4.41. The average distance walked in 6 minutes in the 6MWT was 353.6 meters (SD = 98.5, range: 0 to 583.0).

### Goodness-of-fit test of the initial measurement models and modified measurement models

No high correlation coefficients were observed except between comfortable walking speed and fast walking speed (r = 0.83) (see S1 Table). The variance of continuous variables can be calculated through taking the square of the SD in Table 1. The variances of OW measured by accelerometry + GPS, walking endurance, general mental health, balance capacity, and residential density were more than 500 times of that of comfortable and fast walking speed. Therefore, to prevent the ill-scaled covariance matrix problem, OW measured by accelerometry + GPS, walking endurance, general mental health, balance capacity, and residential density were rescaled through dividing by 60, 60, 10, 5, and 100, respectively, while comfortable and fast walking speed were rescaled through multiplying by 10.

**Fig 3:**
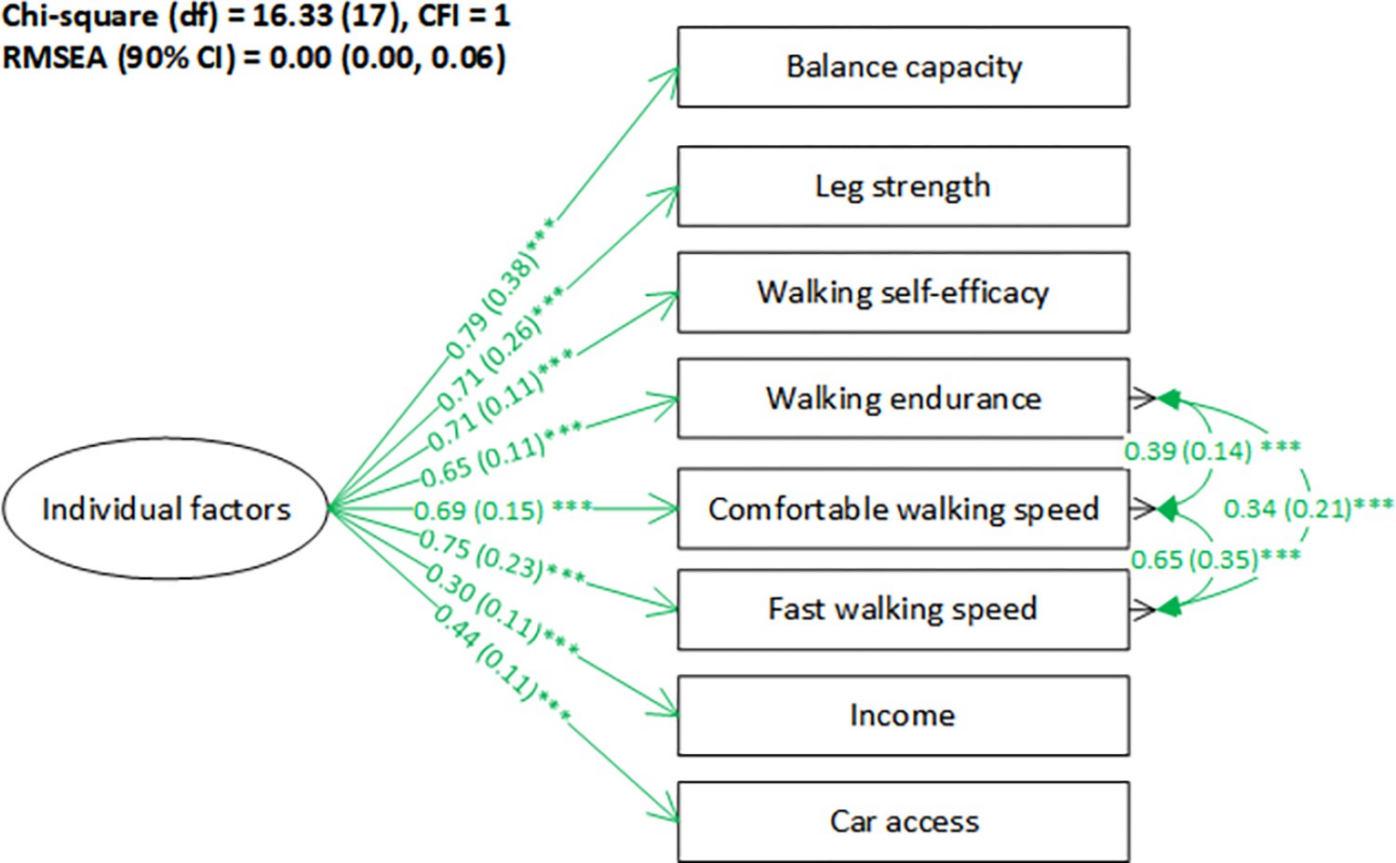
Modified measurement model for the individual factors. Note: The format of factor loadings and covariances: standardized estimation (standard error) ^significance level^. * p < .05; **.01 < p < .05; *** p < .001. The green color indicates a positive relationship while red indicates a negative relationship.

The initial measurement model for the individual factors showed a good model fit (*χ*^2^ = 30.14, df = 30, CFI = 1.00, RMSEA (90%CI) = 0.005 (0.000–0.053)) (see S1 Fig). The covariances among comfortable walking speed, fast walking speed, and walking endurance were added based on their strong correlation coefficient (r ≥ 0.6) [70] and the nature of these measurements. The covariance between general mental health and walking self-efficacy [73, 74], between education and car access [75] are supported by the literature. This model had significantly better fit than the model which added the fear of personal safety as one observed variable for the individual factors (see S2 Fig) with chi-square difference = 18.42, df = 9, p < .05, lower CFI and larger RMSEA. Therefore, we proceeded with the initial measurement model for the individual factors without fear of personal safety. Further, the results indicated that general mental health and education were not significantly related to the individual factors. Thus, a reduced model with these two observed variables removed was tested. This modified measurement model (Fig 3) showed a better fit to the data (*χ*^2^ = 16.33, df = 17, CFI = 1, RMSEA (90% CI) = 0.00 (0.00–0.06)); thus, we proceeded with the reduced model.

**Fig 4:**
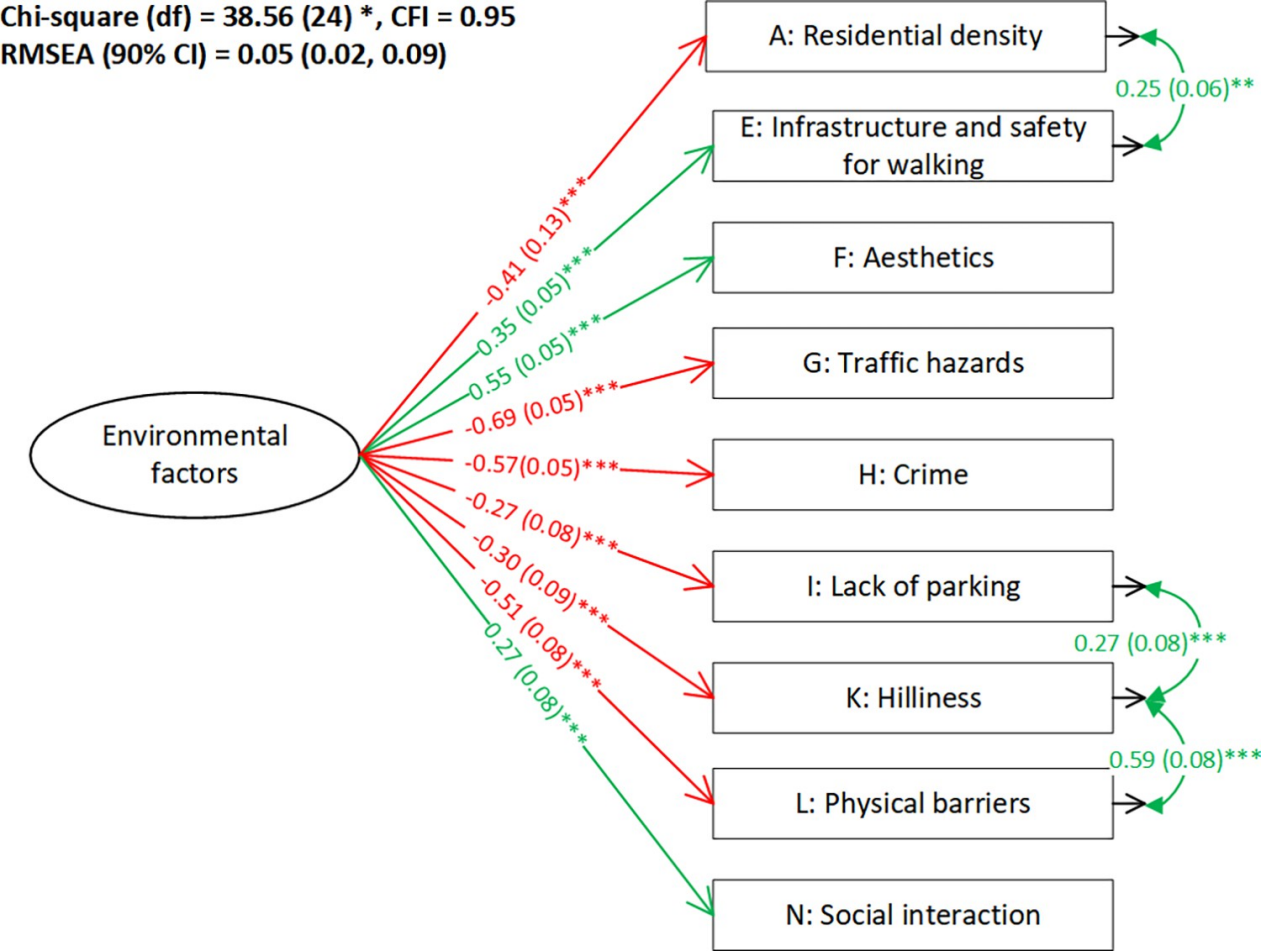
Modified measurement model for the environmental factors. Note: The format of factor loadings and covariances: standardized estimation (standard error) ^significance level^. * p < .05; **.01 < p < .05; *** p < .001. The green color indicates a positive relationship while red indicates a negative relationship. A: higher scores indicate higher density; E: higher scores indicate more infrastructure and safer neighborhood; F: higher scores indicate more aesthetically appealing neighborhood; G: higher scores indicate more traffic hazards; H: higher scores indicate higher crime rate and feeling of unsafe to walk in the neighborhood; I: higher scores indicate that parking is more difficult in local shopping areas; K: higher scores indicate that the streets in the neighborhood is more hilly; L: higher scores indicate more physical barriers to walking in the neighborhood; N: higher scores indicate more social interactions while walking in the neighborhood.

The initial measurement model for the environmental factors showed acceptable goodness-of-fit (*χ*^2^ = 76.30, df = 51, CFI = 0.93, RMSEA (90% CI) = 0.05 (0.02–0.07)) (see S3 Fig). The covariances among the composite subscale A to subscale H were consistent with the measurement model for confirming the validity of the NEWS questionnaire [19, 20]. Non-significant observed variables (subscales B (land use mix diversity), C (land use mix access), D (street connectivity), and J (lack of cul-de-sacs)) were removed. The modified measurement model (Fig 4) presented good model fit (*χ*^2^ = 38.56, df = 24, CFI = 0.95, RMSEA (90% CI) = 0.05 (0.02–0.09)). The covariance between hilliness and physical barriers was implied by their definitions, as hilly streets can be considered as one type of physical barrier. The covariance between hilliness and lack of parking was implied by the strong correlation coefficients (r = 0.64) [70].

### Results of SEM analysis

**Fig 5:**
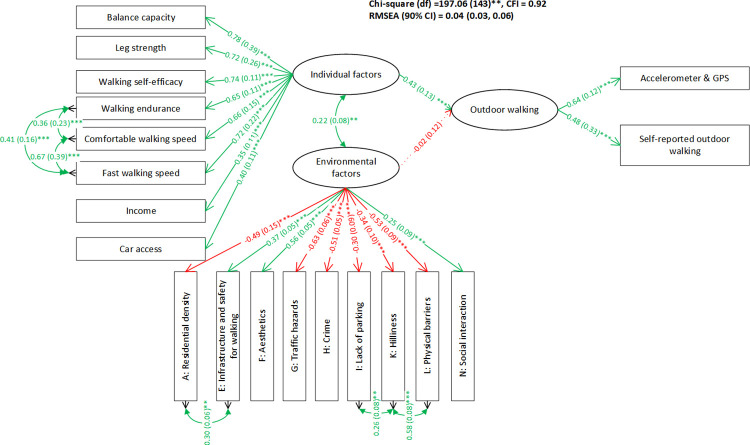
The SEM for outdoor walking. Note: The format of factor loadings and covariances: standardized estimation (standard error) ^significance level^; the format of path coefficients: unstandardized estimation (standard error) ^significance level^. * p < .05; ** .01 < *p* < .05; *** *p* < .001. The green color indicates a positive relationship while red color indicates a negative relationship.

The SEM built on the modified measurement models for the individual factors and environmental factors presented acceptable goodness-of-fit (*χ*^2^ = 197.06, df = 143, CFI = 0.92, RMSEA (90% CI) = 0.04 (0.03–0.06)) (See Fig 5). The latent construct for individual factors was significantly related to OW (p < .001). The standardized factor loadings from the individual factors on balance capacity, fast walking speed, walking self-efficacy, leg strength, walking endurance and comfortable walking speed were all above 0.65 while those on car access and income were 0.40 and 0.35 respectively. The factor loadings on all the observed variables were positive, so that this latent construct represented higher levels of the individual factors. The positive path coefficient indicated that a one unit increase in the individual factors was associated with a 0.43-unit increase in OW. The latent construct of environmental factors was not significantly related to OW (p = 0.88) and the estimated path coefficient was close to zero (-0.02). This latent construct represented higher levels of walkability in the environmental factors. This is because: a) a higher score on infrastructure and safety for walking, aesthetics, and social interaction indicates a better condition and their factor loadings were positive; b) a higher score on residential density, traffic hazards, crime, lack of parking, hilliness and physical barriers indicates a worse condition and their factor loadings were negative. The covariance of the two latent constructs–individual and environmental factors–was significant and positive (r = 0.22, p < 0.01).

This SEM had similar goodness-of-fit compared to the model that only treated the device-based OW measurement as the primary outcome (see S4 Fig) with chi-square difference equals 13.44, df = 16, p = 0.64. The parameter estimations were very similar except the path coefficient from individual factors to OW (0.43 vs 0.24). The sensitivity analysis showed that the parameter estimations of the SEM when data from 11 participants who performed the 6MWT using a 10-meter walkway instead of the 30-meter walkway were excluded (see S5 Fig) were almost the same as the estimations in Fig 5 presenting the results using data on all 205 participants. See S1 File for the author-generated R code used in the analysis.

## Discussion

This study aimed to estimate the extent to which individual variables, environmental variables, and OW inter-relate as depicted in the GO-OUT conceptual model. Findings revealed that individual variables that reflect walking capacity (i.e., comfortable and fast walking speed, walking endurance, leg strength, balance capacity, walking self-efficacy), income, and car access, positively correlated with OW of community-dwelling older adults with self-reported difficulties walking outdoors. This result is consistent with a previous report [76] indicating that physical limitations, such as difficulties in climbing stairs and walking, and fear of falling, negatively affect the odds of going outdoors in Swiss community-dwelling older adults. Likewise, another study found that higher values for total daily steps walked was associated with faster walking speed, more repetitions of timed chair stands and greater grip strength in American adults ≥65 years of age [77]. Measures of walking speed, walking endurance, leg strength, and balance ability are commonly used clinically and in research to assess walking ability in older adults [78–82]. Among them, walking speed is also often used as an overall measure of preparedness for safe community mobility [83, 84], further supporting the positive relationships between the individual variables related to walking capacity and OW. Walking self-efficacy, which refers to older adults’ confidence in walking outdoors, should predict OW behaviour based on self-efficacy theory [85] and has been shown to be significantly related to physical activity in several studies [22, 86, 87]. Individuals with higher income have been shown to have higher levels of mobility [88]. Moreover, positive associations between having access to a vehicle and outdoor physical activities were shown in previous studies [89].

Our findings demonstrated that environmental variables were not significantly associated with OW. This finding is not consistent with some studies (i.e., a review paper focussed on physical activity, and studies that measured OW by self-report questionnaire) that showed the built environment could promote outdoor activities among older adults [66, 90–92]. On the other hand, one longitudinal study found that changes in neighbourhood walkability were not significantly associated with changes in walking activity in older adults [93]. Similar to the current study, a study conducted in Vancouver, Canada did not show significant relationships between neighbourhood walkability and physical activity in older adults [94]. Participants in the Vancouver study had a similar age distribution to the current study sample, although the Vancouver sample was from a low-income population [94]. Another study on a large sample of Canadian adults (mean age of 47 years) [95] also found no significant association between daily steps and neighbourhood walkability.

Results from the current study demonstrated environmental variables were related to OW indirectly through the individual factors. This highlights the importance of infrastructure and urban planning to promote outdoor walking in community-dwelling older adults with self-reported difficulties walking outdoors. This is consistent with findings from previous research [23] that environmental factors have indirect influences on physical activity through self-efficacy, and perceived pros and cons of physical activity. However, the individuals in that project were younger (i.e., mean age 44 years) than participants in our study (i.e., mean age 74 years) [23]. The positive association between the individual factors and the environmental factors is supported by literature that determined more walkable neighbourhoods were associated with better physical function [96]. Other studies have identified that people with higher income live in more walkable neighbourhoods [97, 98].

This study had several limitations. First, participants in this study were older adults living in urban centres who self-reported having difficulty walking outdoors. In addition, 73% of the study population were women. Thus, caution is needed when generalizing the results from the current study to the general population of older adults. Moreover, the study sample was predominated by individuals with high education levels (62% had completed college or university programs). This may be one reason why education was not significant as an observed variable for the individual factors. The perceived environment was used in this study; however, objective measurements of built environment could be adopted in future studies. The sample size was relatively small with 205 participants, however, there is little consensus on the adequate sample size for sufficient statistical power when using SEM [99, 100]. Our sample size was adequate using the 10 observations per indicator rule [101] as we had 19 observed variables. Our relatively small sample size was one reason for removing non-significant observed variables in the initial measurement models for individual and environmental factors [67, 68]. Besides, data on other environmental variables such as temperature, cloud coverage and wind that have been shown to be related to outdoor physical activities [102] were not included. Furthermore, study data were collected in spring months in Canada, which could limit the generalizability of results from the current study to other times of the year. However, a strength with the timing of data collection being in the spring when weather is moderate, is that it may actually increase the generalizability of our findings for other regions with moderate temperatures. Although the participants were recruited from four sites across Canada to make the study population representative of Canadian older adults with self-reported difficulties walking outdoors, geographical factors such as biodiversity in natural areas were not considered in this study. Therefore, caution needs to be taken when generalising the results of the current study to other countries that have different geographical features.

Physiotherapists often work with community dwelling older adults who have difficulties in walking outdoors and have personal goals of increasing community ambulation. Environmental factors had an indirect association with outdoor walking through individual factors, highlighting the importance of considering environmental factors when addressing walking in a community setting. Physiotherapists should consider interventions that address individual factors associated with outdoor walking and integrate practice in relevant environments. For example, a walking program could focus on outdoor walking endurance and integrate walking up hills. Assessment of aspects of the individual factors such as balance, leg strength, endurance, walking speed and self-efficacy may help physiotherapists to identify areas in which to focus intervention strategies to attain the client’s goal of improving community ambulation or OW. Income of the client and access to a car or other transportation need to be considered when assisting a client in determining safe walking locations in the community. Other environmental factors, such as aesthetics, crime and social interactions will also assist the physiotherapist and client in choosing walking locations likely to lead to attainment of their OW goals.

## Conclusions

Reduced OW is associated with lower walking capacity and lower confidence in walking outdoors in older adults with self-reported walking outdoors limitations. Better neighbourhood walkability may indirectly contribute to more OW. The visual depiction of relationships between individual and environmental variables and OW should facilitate understanding of the model by researchers, clinicians, and the public. The findings in this study can be used to guide the design of interventions targeting OW in older adults and community-based clinical practice.

## Supporting information

S1 Fig Initial measurement model for the individual factors. (TIF)

S2 Fig The measurement model for the individual factors including fear of personal safety measured by crime from the NEWS. (TIF)

S3 Fig Initial measurement model for the environmental factors. Note: A: higher scores indicate higher density; B: higher scores indicate higher level of land use mix diversity; C: higher scores indicate higher level of land use mix access; D: higher scores indicate more street connectivity; E: higher scores indicate more infrastructure and safer neighborhood; F: higher scores indicate more aesthetically appealing neighborhood; G: higher scores indicate more traffic hazards; H: higher scores indicate higher crime rate and feeling of unsafe to walk in the neighborhood; I: higher scores indicate that parking is more difficult in local shopping areas; J: higher scores indicate lack of cul-de-sacs; K: higher scores indicate that the streets in the neighborhood is more hilly; L: higher scores indicate more physical barriers to walking in the neighborhood; N: higher scores indicate more social interactions while walking in the neighborhood.
(TIF)

S4 Fig The adapted SEM based on the GO-OUT conceptual framework for outdoor walking using device-based measurement. (TIF)

S5 Fig Structural equation model using data excluding participants who had the 6-minute test with a 10-meter walkway instead of the 30-meter walkway in the protocol. Note: The format of factor loadings and covariances: standardized estimation (standard error) significance level; the format of path coefficients: unstandardized estimation (standard error) significance level. * p < .05; ** .01 < p < .05; *** p < .001. The green color indicates a positive relationship while red color indicates a negative relationship.
(TIF)

S1 File R code. (DOCX)

S1 Table Correlation coefficient matrix of all variables. (DOCX)

## List of abbreviations

ASCQ: Ambulatory Self-Confidence Questionnaire
CFI: Comparative Fit Index
CHAMPS: Community Health Activities Model Program for Seniors
CHAMPS-OUTDOORS: Community Health Activities Model Program for Seniors Outdoor Walking
CI: Confidence Interval
GO-OUT: Getting Older Adults Outdoors
GPS: Global Positioning System
ICC: Intraclass Correlation Coefficient
mini-BESTest: Mini Balance Evaluation Systems Test
NEWS: Neighbourhood Environment Walkability Scale
OW: Outdoor Walking
RAND-36: Research ANd Development-36
RMSEA: Root Mean Square Error of Approximation
SEM: Structural Equation Modeling
SD: Standard Deviation
WLSMV: Weighted Least Square Mean and Variance
6MWT: 6-Minute Walk Test
