# Supplementary figures and images for: Individual and environmental variables related to outdoor walking among older adults: Verifying a model to guide the design of interventions targeting outdoor walking
Source: PLoS One. 2024 Jan 10;19(1):e0296216. doi: 10.1371/journal.pone.0296216 (PMC10781134; doi:10.1371/journal.pone.0296216)

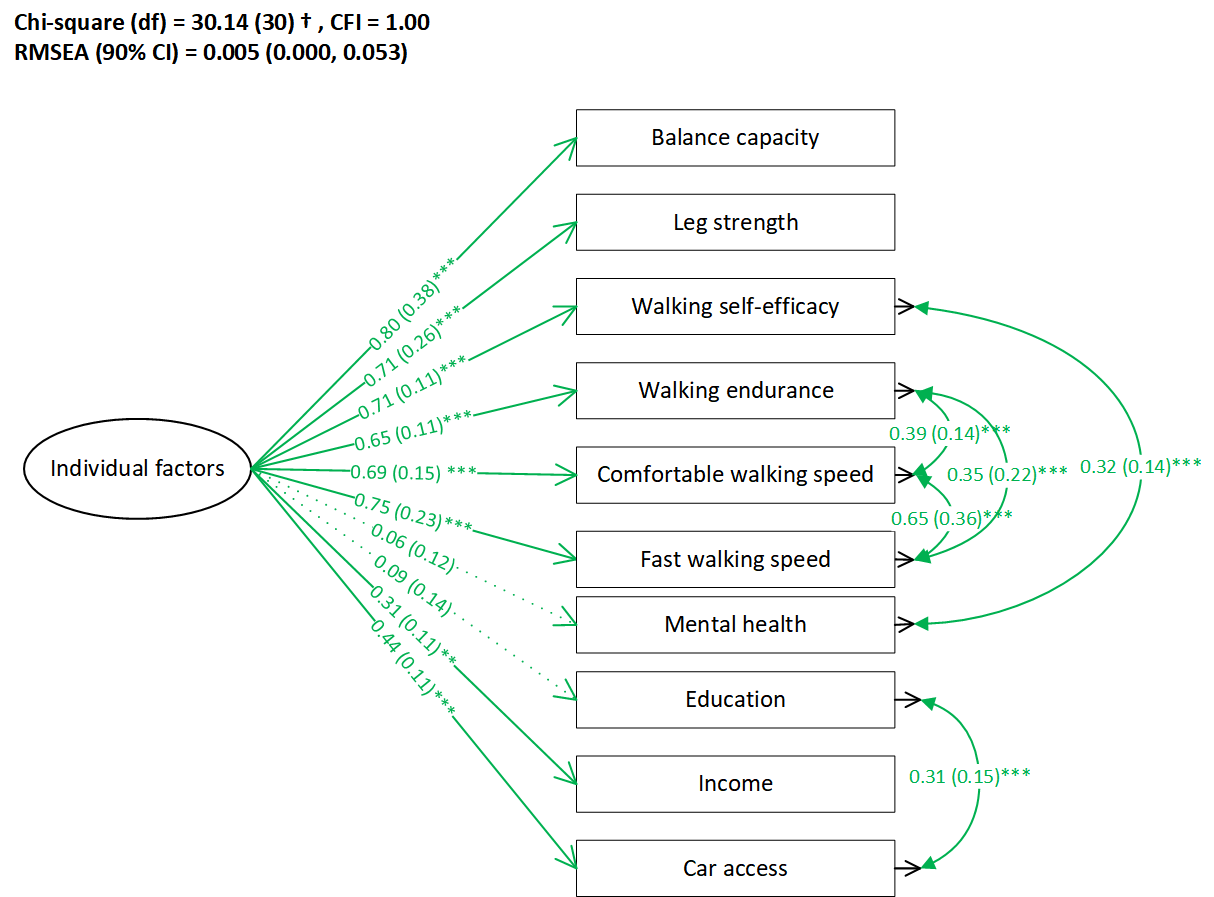

Supplement: S1 Fig — (TIF) [file pone.0296216.s001.tif]

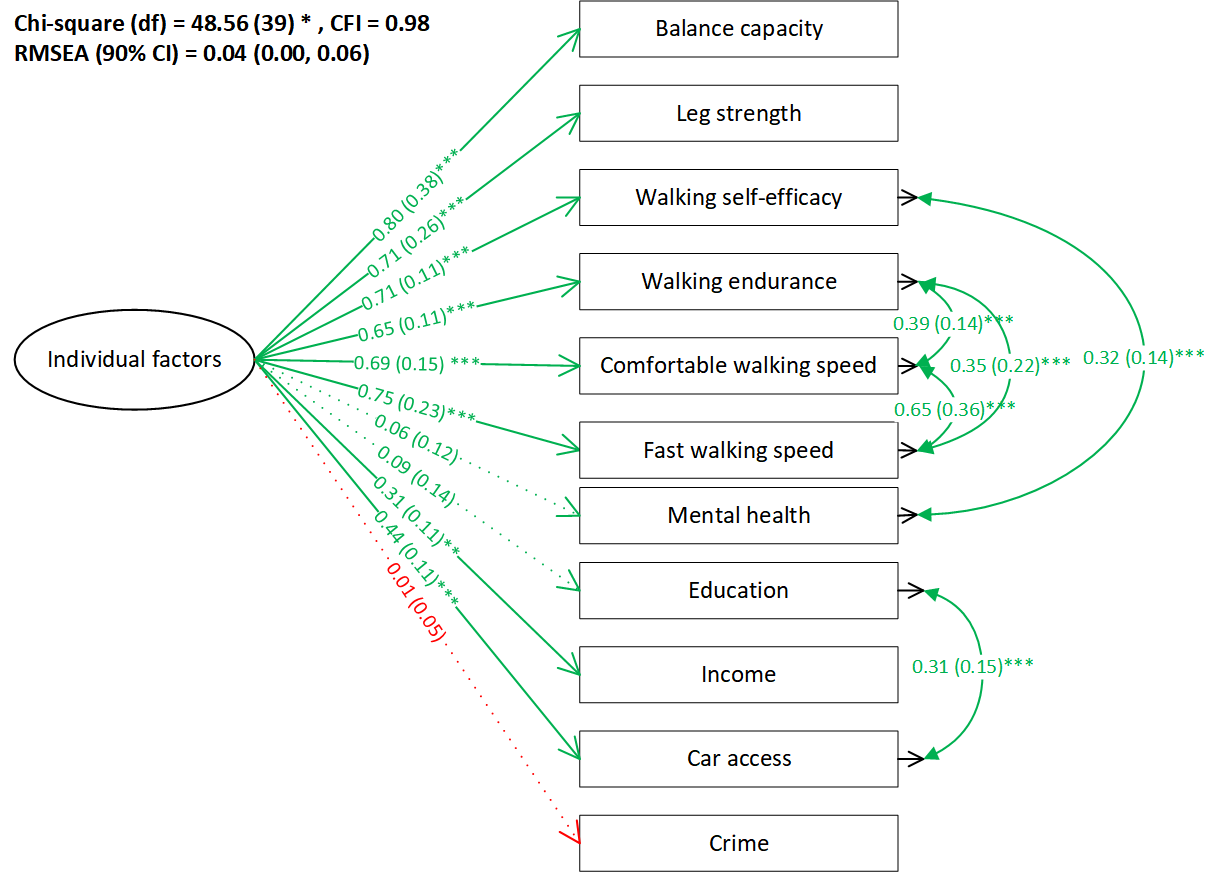

Supplement: S2 Fig — (TIF) [file pone.0296216.s002.tif]

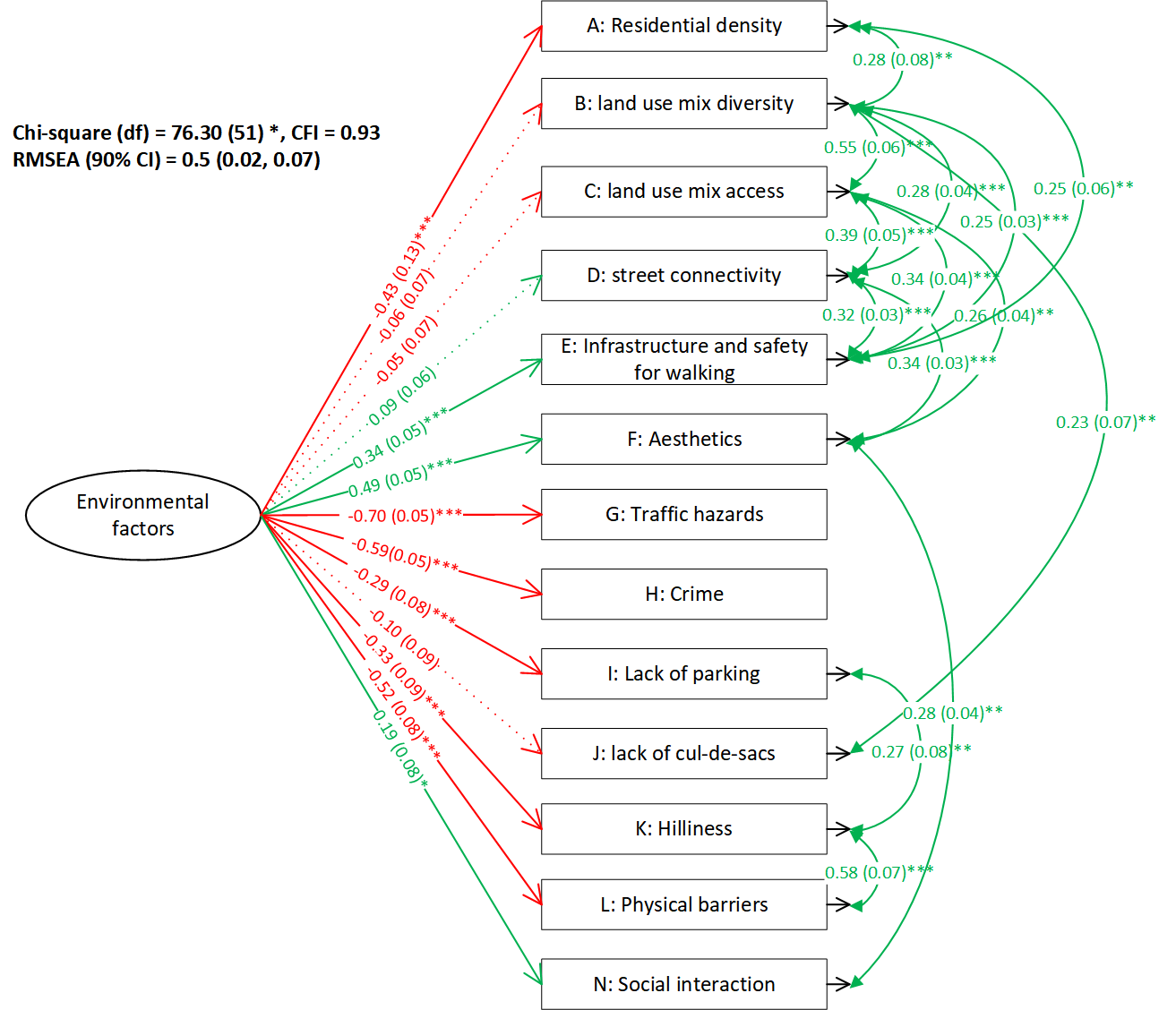

Supplement: S3 Fig — Note: A: higher scores indicate higher density; B: higher scores indicate higher level of land use mix diversity; C: higher scores indicate higher level of land use mix access; D: higher scores indicate more street connectivity; E: higher scores indicate more infrastructure and safer neighborhood; F: higher scores indicate more aesthetically appealing neighborhood; G: higher scores indicate more traffic hazards; H: higher scores indicate higher crime rate and feeling of unsafe to walk in the neighborhood; I: higher scores indicate that parking is more difficult in local shopping areas; J: higher scores indicate lack of cul-de-sacs; K: higher scores indicate that the streets in the neighborhood is more hilly; L: higher scores indicate more physical barriers to walking in the neighborhood; N: higher scores indicate more social interactions while walking in the neighborhood. (TIF) [file pone.0296216.s003.tif]

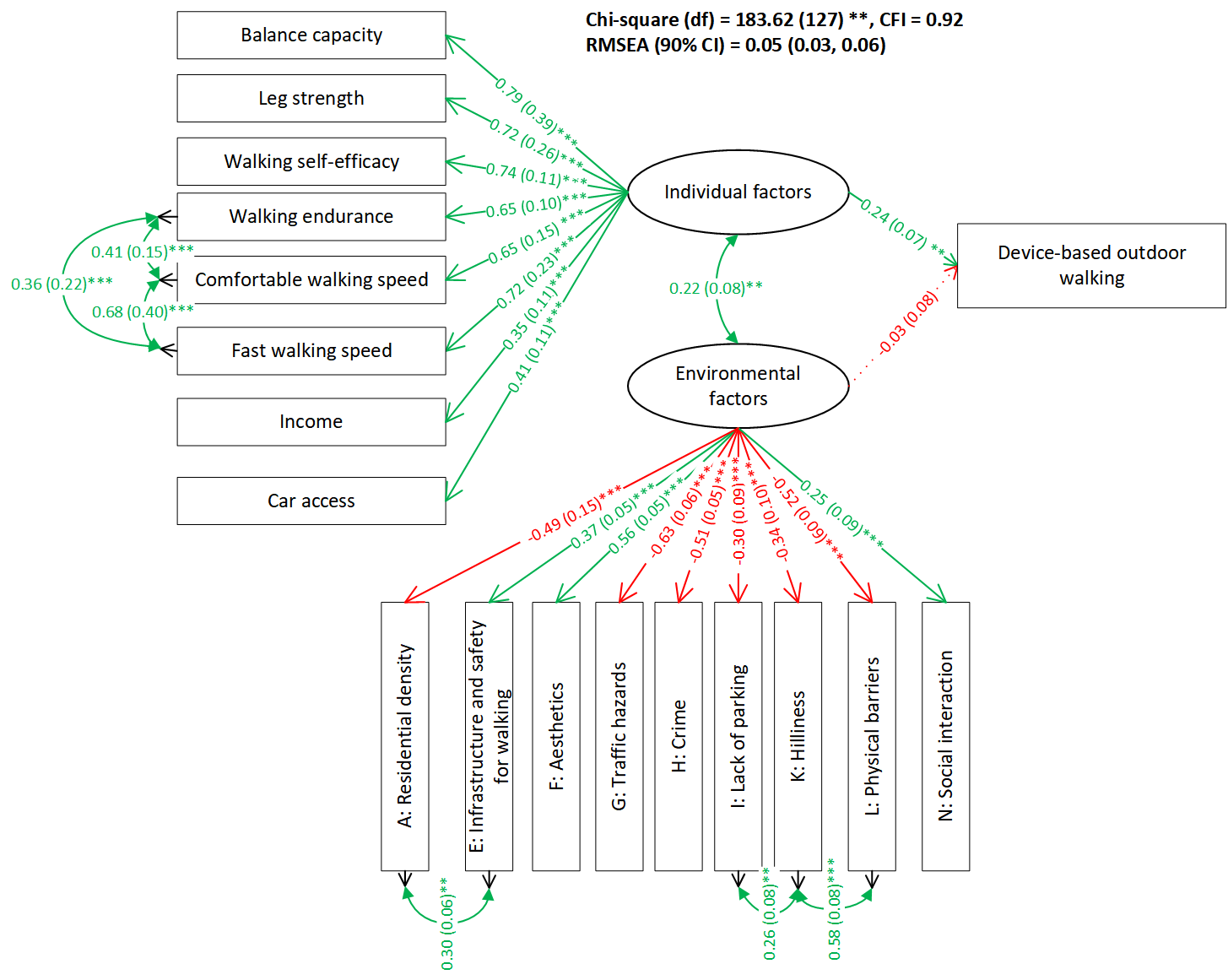

Supplement: S4 Fig — (TIF) [file pone.0296216.s004.tif]

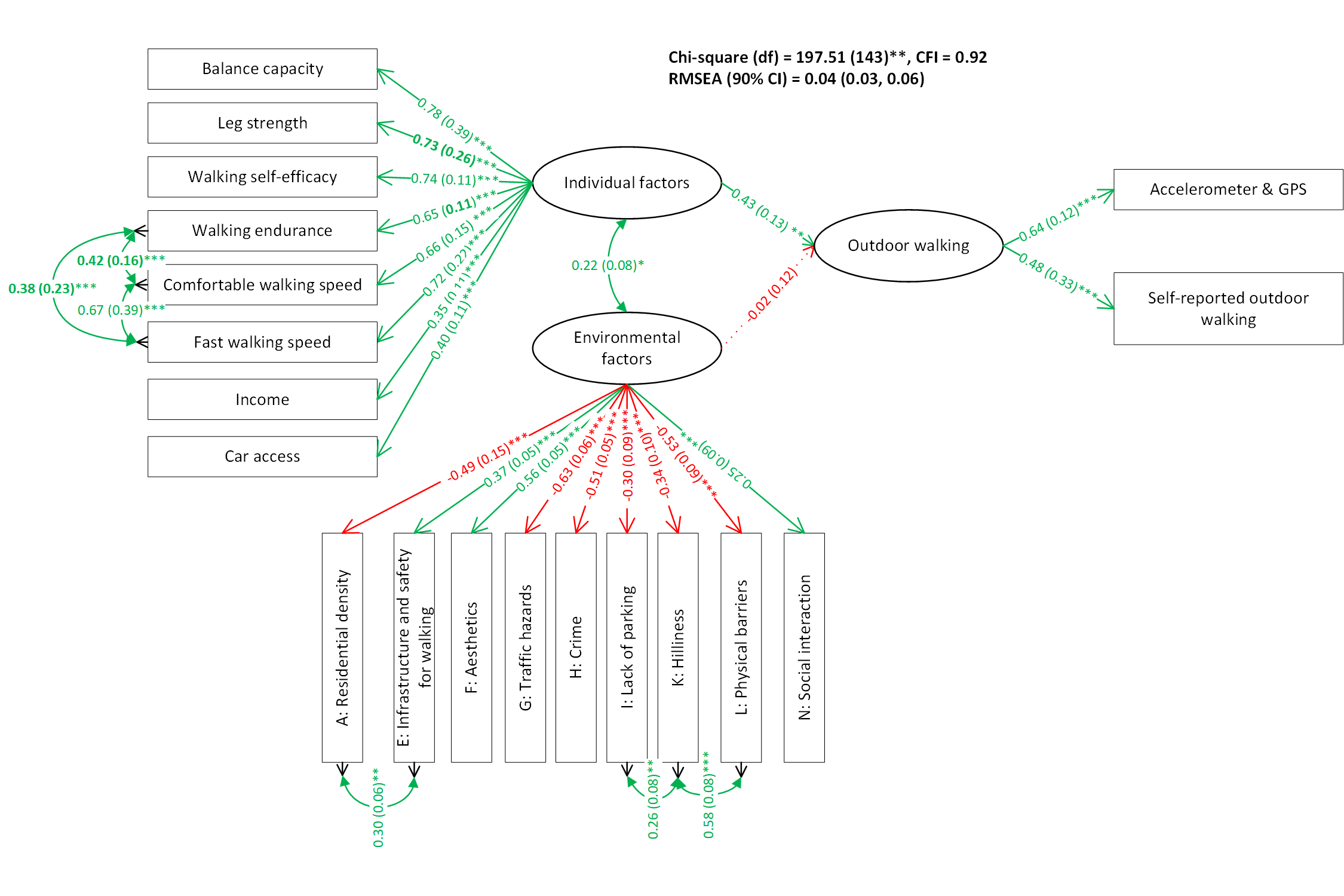

Supplement: S5 Fig — Note: The format of factor loadings and covariances: standardized estimation (standard error) significance level; the format of path coefficients: unstandardized estimation (standard error) significance level. * p < .05; ** .01 < p < .05; *** p < .001. The green color indicates a positive relationship while red color indicates a negative relationship. (TIF) [file pone.0296216.s005.tif]
