# Supplementary material for: Individual and environmental variables related to outdoor walking among older adults: Verifying a model to guide the design of interventions targeting outdoor walking
Source: PLoS One. 2024 Jan 10;19(1):e0296216. doi: 10.1371/journal.pone.0296216 (PMC10781134; doi:10.1371/journal.pone.0296216)
Supplement: S1 File — (DOCX) [file pone.0296216.s006.docx]

**Main R code used in the manuscript “Individual and environmental variables related to outdoor walking among older adults: verifying a model to guide the design of interventions targeting outdoor walking”**

# libraries

library(tidyverse)

library(lavaan)

# load the data

baseline <- read_csv("data/baseline.csv")

# treat those indicators as ordered categorical indicators

names <- c("news_sub_i", "news_sub_j", "news_sub_k", "news_sub_l", "news_sub_n")

baseline[,names] <- lapply(baseline[,names] , ordered)

# combine the "Not enough to make ends meet" and "Just enough to make ends meet" as the "not

# enough to make ends meet" only has 3 observations

baseline$income <- ifelse(baseline$income %in% c(1,2), 0, ifelse(is.na(baseline$income),NA, 1))

baseline$income <- ordered(baseline$income)

# For education, combining:

# less than primary + completed primary + less than secondary into 1 category – ‘less than secondary’

# Some college + some university to ‘some post secondary’

baseline$edu_cat <- ifelse(baseline$edu %in% c("Less than primary (Kindergarten - grade 6)",

"Completed primary (Kindergarten - grade 6)",

"Less than secondary (grade 9-12)"), "less than secondary",

ifelse(baseline$edu %in% c("Some university courses",

"Some College courses (In Quebec - CEGEP)"), "some post secondary",

baseline$edu

))

baseline$edu_cat <- factor(baseline$edu_cat, levels = c("less than secondary","some post secondary", "Completed secondary (grade 9-12)",

"Completed College (In Quebec - CEGEP)",

"Completed university (bachelor degree)","Graduate program (e.g. MSc, PhD)"))

baseline$edu_cat_num <- as.numeric(baseline$edu_cat)

# car access

baseline$Car_access <- ifelse(baseline$Car_access == "Yes", 1, 0)

names <- c("edu_cat", "Car_access")

baseline[,names] <- lapply(baseline[,names] , ordered)

# ill scaling check

var_check_data <- baseline %>%

select(champs_ow_total, Min.walked.outside..MP.CS.7.days.,

walk_10_meters_comfortable_pace, walk_10_meters_fast_walking_speed, mwt6_total_raw,

rand_em, ascq, sts30_raw, Minibest_total, edu_cat_num,

news_sub_a, news_sub_b, news_sub_c, news_sub_d, news_sub_e, news_sub_f, news_sub_g,

news_sub_h)

var_checking <- c()

for (i in 1:21) {

var_checking[i] <- var(var_check_data[,i], na.rm=TRUE)

}

var_checking

# rescale

baseline <- baseline %>%

mutate(mwt6_total_raw_s = mwt6_total_raw/60,

walk_10_meters_comfortable_pace_s = walk_10_meters_comfortable_pace*10,

walk_10_meters_fast_walking_speed_s = walk_10_meters_fast_walking_speed*10,

rand_em_s = rand_em/10,

Minibest_total_s = Minibest_total/5 )%>%

mutate(news_sub_a_s = news_sub_a/100)%>%

mutate(Min.walked.outside..MP.CS.7.days._s = Min.walked.outside..MP.CS.7.days./60)

var_check_data <- baseline %>%

select(champs_ow_total, Min.walked.outside..MP.CS.7.days._s,

walk_10_meters_comfortable_pace_s, walk_10_meters_fast_walking_speed_s, mwt6_total_raw_s,

rand_em_s, ascq, sts30_raw,

Minibest_total_s, edu_cat_num,

news_sub_a_s, news_sub_b, news_sub_c, news_sub_d, news_sub_e, news_sub_f, news_sub_g, news_sub_h)

var_checking <- c()

for (i in 1:18) {

var_checking[i] <- var(var_check_data[,i], na.rm=TRUE)

}

var_checking

##############################################

# start the modeling for measurement models for the indivudal and environmental factor

# the initial model for individual factors

model_ind_1 <- 'ind =~

walk_10_meters_comfortable_pace_s + walk_10_meters_fast_walking_speed_s +

mwt6_total_raw_s + rand_em_s + ascq + sts30_raw + Minibest_total + edu_cat_num +

Car_access + income

# covariance

walk_10_meters_comfortable_pace_s ~~ walk_10_meters_fast_walking_speed_s

walk_10_meters_comfortable_pace_s ~~ mwt6_total_raw_s

walk_10_meters_fast_walking_speed_s ~~ mwt6_total_raw_s

rand_em_s ~~ ascq

Car_access ~~ edu_cat_num

'

model_ind_1_fit <- cfa(model_ind_1, data = baseline, missing = "pairwise", parameterization = "delta", estimator="WLSMV", std.lv = TRUE)

summary(model_ind_1_fit, fit.measures = TRUE, standardized = TRUE)

# the individual factor includes fear of walking in their neighbourhood (crime (subscale h))

model_ind_1_includeCrime <- 'ind =~

walk_10_meters_comfortable_pace_s + walk_10_meters_fast_walking_speed_s +

mwt6_total_raw_s + rand_em_s + ascq + sts30_raw + Minibest_total + edu_cat_num +

Car_access + income + news_sub_h

# covariance

walk_10_meters_comfortable_pace_s ~~ walk_10_meters_fast_walking_speed_s

walk_10_meters_comfortable_pace_s ~~ mwt6_total_raw_s

walk_10_meters_fast_walking_speed_s ~~ mwt6_total_raw_s

rand_em_s ~~ ascq

Car_access ~~ edu_cat_num

'

model_ind_1_includeCrime_fit <- cfa(model_ind_1_includeCrime, data = baseline, missing = "pairwise", parameterization = "delta", estimator="WLSMV", std.lv = TRUE)

summary(model_ind_1_includeCrime_fit, fit.measures = TRUE, standardized = TRUE)

# the modified model for individual factors (removed general mental health and education)

model_ind_modified <- 'ind =~

walk_10_meters_comfortable_pace_s + walk_10_meters_fast_walking_speed_s +

mwt6_total_raw_s + ascq + sts30_raw + Minibest_total + income + Car_access

# covariance

walk_10_meters_comfortable_pace_s ~~ walk_10_meters_fast_walking_speed_s

walk_10_meters_comfortable_pace_s ~~ mwt6_total_raw_s

walk_10_meters_fast_walking_speed_s ~~ mwt6_total_raw_s

'

model_ind_modified_fit <- cfa(model_ind_modified, data = baseline, missing = "pairwise", parameterization = "delta", estimator="WLSMV", std.lv = TRUE)

summary(model_ind_modified_fit, fit.measures = TRUE, standardized = TRUE)

# the initial CFA for environmental factors

model_env_1 <- 'env =~ news_sub_d +

news_sub_e + news_sub_f + news_sub_g + news_sub_h + news_sub_b + news_sub_c +

news_sub_i + news_sub_j + news_sub_k + news_sub_l + news_sub_n + news_sub_a_s

# covariance

news_sub_b ~~ news_sub_c

news_sub_k ~~ news_sub_l

news_sub_c ~~ news_sub_d

news_sub_c ~~ news_sub_e

news_sub_b ~~ news_sub_d

news_sub_b ~~ news_sub_e

news_sub_d ~~ news_sub_e

news_sub_d ~~ news_sub_f

news_sub_a_s ~~ news_sub_b

news_sub_i ~~ news_sub_k

news_sub_a_s ~~ news_sub_e

news_sub_f ~~ news_sub_n

news_sub_b ~~ news_sub_j

news_sub_c ~~ news_sub_f

'

model_env_1_fit <- cfa(model_env_1, data = baseline, missing = "pairwise", parameterization = "delta", estimator="WLSMV", std.lv = TRUE)

summary(model_env_1_fit, fit.measures = TRUE, standardized = TRUE)

# the modified model for individual factors

model_env_modified <- 'env =~ news_sub_e + news_sub_f + news_sub_g + news_sub_h + news_sub_i +

news_sub_k + news_sub_l + news_sub_n + news_sub_a_s

# covariance

news_sub_k ~~ news_sub_l

news_sub_a_s ~~ news_sub_e

news_sub_i ~~ news_sub_k

'

model_env_modified_fit <- cfa(model_env_modified, data = baseline, missing = "pairwise", parameterization = "delta", estimator="WLSMV", std.lv = TRUE)

summary(model_env_modified_fit, fit.measures = TRUE, standardized = TRUE)

# SEM built on the modified measurement models

SEM_outdoor_walking <- 'env =~ news_sub_e + news_sub_f + news_sub_g + news_sub_h +

news_sub_i + news_sub_k + news_sub_l + news_sub_n + news_sub_a_s

ind =~ walk_10_meters_comfortable_pace_s + walk_10_meters_fast_walking_speed_s +

mwt6_total_raw_s + ascq + sts30_raw + Minibest_total + income + Car_access

outdoor_walking =~ Min.walked.outside..MP.CS.7.days._s + champs_ow_total

#regression

outdoor_walking ~ env + ind

# covariance

walk_10_meters_comfortable_pace_s ~~ walk_10_meters_fast_walking_speed_s

walk_10_meters_comfortable_pace_s ~~ mwt6_total_raw_s

walk_10_meters_fast_walking_speed_s ~~ mwt6_total_raw_s

news_sub_k ~~ news_sub_l

news_sub_a_s ~~ news_sub_e

news_sub_i ~~ news_sub_k

'

SEM_outdoor_walking_fit <- sem(SEM_outdoor_walking, data = baseline, missing = "pairwise", parameterization = "delta", estimator="WLSMV", std.lv = TRUE)

summary(SEM_outdoor_walking_fit, fit.measures = TRUE, standardized = TRUE)

# SEM only treat the device-based outdoor walking as the primary outcome

SEM_device_OW <- 'env =~ news_sub_e + news_sub_f + news_sub_g + news_sub_h + news_sub_i + news_sub_k + news_sub_l +

news_sub_n + news_sub_a_s

ind =~ walk_10_meters_comfortable_pace_s + walk_10_meters_fast_walking_speed_s +

mwt6_total_raw_s + ascq + sts30_raw + Minibest_total + income + Car_access

#regression

Min.walked.outside..MP.CS.7.days._s ~ env + ind

# covariance

# env ~~ 0*ind

walk_10_meters_comfortable_pace_s ~~ walk_10_meters_fast_walking_speed_s

walk_10_meters_comfortable_pace_s ~~ mwt6_total_raw_s

walk_10_meters_fast_walking_speed_s ~~ mwt6_total_raw_s

news_sub_k ~~ news_sub_l

news_sub_a_s ~~ news_sub_e

news_sub_i ~~ news_sub_k

'

SEM_device_OW_fit <- sem(SEM_device_OW, data = baseline, missing = "pairwise", parameterization = "delta", estimator="WLSMV", std.lv = TRUE)

summary(SEM_device_OW_fit, fit.measures = TRUE, standardized = TRUE)
