# Supplementary material for: Individual and environmental variables related to outdoor walking among older adults: Verifying a model to guide the design of interventions targeting outdoor walking
Source: PLoS One. 2024 Jan 10;19(1):e0296216. doi: 10.1371/journal.pone.0296216 (PMC10781134; doi:10.1371/journal.pone.0296216)
Supplement: S1 Table — (DOCX) [file pone.0296216.s007.docx]

**S1 Table. Correlation coefficient matrix of all variables**

|  | 1 | 2 | 3 | 4 | 5 | 6 | 7 | 8 | 9 | 10 | 11 | 12 | 13 | 14 | 15 | 16 | 17 | 18 | 19 | 20 | 21 | 22 | 23 | 24 | 25 |
| --- | --- | --- | --- | --- | --- | --- | --- | --- | --- | --- | --- | --- | --- | --- | --- | --- | --- | --- | --- | --- | --- | --- | --- | --- | --- |
| 1. Outdoor walking by CHAMPS | 1 |  |  |  |  |  |  |  |  |  |  |  |  |  |  |  |  |  |  |  |  |  |  |  |  |
| 2. Outdoor walking by accelerometry+GPS | 0.31 | 1.00 |  |  |  |  |  |  |  |  |  |  |  |  |  |  |  |  |  |  |  |  |  |  |  |
| 3. Comfortable walking speed | 0.17 | 0.21 | 1 |  |  |  |  |  |  |  |  |  |  |  |  |  |  |  |  |  |  |  |  |  |  |
| 4. Fast walking speed | 0.13 | 0.22 | **0.83** | 1 |  |  |  |  |  |  |  |  |  |  |  |  |  |  |  |  |  |  |  |  |  |
| 5. Walking endurance | 0.10 | 0.23 | 0.66 | 0.66 | 1 |  |  |  |  |  |  |  |  |  |  |  |  |  |  |  |  |  |  |  |  |
| 6. General mental health | 0.10 | -0.11 | -0.02 | -0.01 | -0.02 | 1 |  |  |  |  |  |  |  |  |  |  |  |  |  |  |  |  |  |  |  |
| 7. Walking self-efficacy | 0.20 | 0.11 | 0.51 | 0.57 | 0.44 | 0.27 | 1 |  |  |  |  |  |  |  |  |  |  |  |  |  |  |  |  |  |  |
| 8. Leg strength | 0.17 | 0.21 | 0.48 | 0.51 | 0.47 | 0.11 | 0.51 | 1 |  |  |  |  |  |  |  |  |  |  |  |  |  |  |  |  |  |
| 9. Balance capacity | 0.09 | 0.16 | 0.53 | 0.59 | 0.57 | 0.11 | 0.52 | 0.59 | 1 |  |  |  |  |  |  |  |  |  |  |  |  |  |  |  |  |
| 10. Income | 0.12 | 0.10 | 0.19 | 0.20 | 0.11 | 0.09 | 0.29 | 0.17 | 0.25 | 1 |  |  |  |  |  |  |  |  |  |  |  |  |  |  |  |
| 11. Car access | -0.15 | -0.31 | 0.32 | 0.33 | 0.22 | 0.07 | 0.31 | 0.31 | 0.34 | 0.29 | 1 |  |  |  |  |  |  |  |  |  |  |  |  |  |  |
| 12. Educational level | 0.03 | -0.04 | 0.11 | 0.06 | 0.03 | -0.04 | 0.06 | 0.02 | 0.09 | 0.16 | 0.32 | 1 |  |  |  |  |  |  |  |  |  |  |  |  |  |
| 13. NEWS subscale A - residential density | -0.05 | 0.08 | -0.08 | -0.14 | -0.11 | -0.05 | -0.27 | -0.19 | -0.14 | -0.21 | -0.29 | 0.06 | 1 |  |  |  |  |  |  |  |  |  |  |  |  |
| 14. NEWS subscale B - land-use mix-diversity | 0.16 | 0.15 | 0.21 | 0.25 | 0.13 | 0.01 | 0.16 | 0.13 | 0.15 | 0.12 | -0.01 | 0.06 | 0.28 | 1 |  |  |  |  |  |  |  |  |  |  |  |
| 15. NEWS subscale C - land-use access | 0.20 | 0.17 | 0.20 | 0.20 | 0.21 | 0.11 | 0.19 | 0.12 | 0.15 | 0.16 | -0.15 | 0.06 | 0.12 | 0.55 | 1 |  |  |  |  |  |  |  |  |  |  |
| 16. NEWS subscale D - street connectivity | 0.05 | 0.02 | 0.13 | 0.10 | 0.15 | 0.03 | 0.13 | 0.07 | 0.08 | 0.00 | 0.03 | -0.00 | 0.03 | 0.28 | 0.39 | 1 |  |  |  |  |  |  |  |  |  |
| 17. NEWS subscale E - infrastructure and safety for walking | -0.05 | 0.17 | 0.02 | 0.08 | 0.05 | 0.12 | 0.12 | -0.01 | 0.07 | 0.16 | -0.10 | -0.09 | 0.06 | 0.21 | 0.31 | 0.33 | 1 |  |  |  |  |  |  |  |  |
| 18. NEWS subscale F - aesthetics | 0.06 | 0.02 | 0.05 | 0.08 | 0.13 | 0.09 | 0.18 | 0.09 | 0.08 | 0.31 | 0.02 | 0.11 | -0.22 | 0.10 | 0.20 | 0.34 | 0.31 | 1 |  |  |  |  |  |  |  |
| 19. NEWS subscale G - traffic hazards | 0.03 | -0.07 | 0.10 | 0.04 | -0.01 | -0.12 | -0.14 | -0.14 | -0.05 | -0.23 | -0.02 | -0.03 | 0.32 | 0.03 | 0.04 | -0.08 | -0.25 | -0.32 | 1 |  |  |  |  |  |  |
| 20. NEWS subscale H - crime | 0.01 | 0.00 | 0.05 | 0.01 | -0.00 | 0.08 | -0.08 | 0.05 | -0.02 | -0.27 | 0.11 | -0.02 | 0.29 | 0.10 | 0.03 | -0.02 | -0.09 | -0.29 | 0.42 | 1 |  |  |  |  |  |
| 21. NEWS subscale I - lack of parking | -0.03 | -0.04 | -0.01 | -0.00 | -0.11 | -0.20 | -0.03 | -0.14 | -0.14 | -0.34 | -0.17 | 0.06 | 0.12 | 0.20 | 0.13 | 0.11 | -0.02 | -0.06 | 0.19 | 0.18 | 1 |  |  |  |  |
| 22. NEWS subscale J - lack of cul-de-sacs | -0.01 | -0.00 | -0.00 | 0.00 | -0.05 | 0.16 | 0.03 | 0.02 | 0.00 | 0.09 | -0.26 | 0.05 | 0.16 | 0.24 | 0.07 | 0.05 | 0.13 | 0.07 | 0.01 | 0.16 | 0.14 | 1 |  |  |  |
| 23. NEWS subscale K - hilliness | -0.05 | -0.01 | -0.02 | -0.01 | -0.20 | -0.17 | -0.18 | -0.07 | -0.18 | -0.01 | -0.04 | 0.04 | 0.11 | 0.10 | 0.03 | -0.10 | -0.08 | -0.10 | 0.31 | 0.18 | 0.33 | 0.06 | 1 |  |  |
| 24. NEWS subscale l - physical barriers | -0.10 | 0.00 | -0.01 | 0.01 | -0.09 | -0.25 | -0.23 | -0.02 | -0.19 | -0.11 | -0.09 | 0.02 | 0.15 | 0.01 | -0.16 | -0.15 | -0.17 | -0.26 | 0.30 | 0.35 | 0.29 | 0.11 | 0.64 | 1 |  |
| 25. NEWS subscale N - social interaction while walking | 0.06 | -0.07 | -0.07 | -0.02 | 0.01 | 0.26 | 0.07 | 0.01 | -0.03 | 0.11 | 0.04 | -0.07 | -0.05 | 0.00 | 0.13 | 0.12 | 0.16 | 0.33 | -0.16 | -0.07 | -0.06 | -0.01 | 0.06 | -0.15 | 1 |
